# The tight Second Law inequality for coherent quantum systems and finite-size heat baths

**DOI:** 10.1038/s41467-021-21140-4

**Published:** 2021-02-10

**Authors:** Marcin Łobejko

**Affiliations:** Institute of Theoretical Physics and Astrophysics, Faculty of Mathematics, Physics and Informatics, University of Gdańsk, 80-308 Gdańsk, Poland

**Keywords:** Quantum mechanics, Theoretical physics, Thermodynamics

## Abstract

In classical thermodynamics, the optimal work is given by the free energy difference, what according to the result of Skrzypczyk et al. can be generalized for individual quantum systems. The saturation of this bound, however, requires an infinite bath and ideal energy storage that is able to extract work from coherences. Here we present the tight Second Law inequality, defined in terms of the ergotropy (rather than free energy), that incorporates both of those important microscopic effects – the locked energy in coherences and the locked energy due to the finite-size bath. The former is solely quantified by the so-called control-marginal state, whereas the latter is given by the free energy difference between the global passive state and the equilibrium state. Furthermore, we discuss the thermodynamic limit where the finite-size bath correction vanishes, and the locked energy in coherences takes the form of the entropy difference. We supplement our results by numerical simulations for the heat bath given by the collection of qubits and the Gaussian model of the work reservoir.

## Abstract (web summary)

Quantum versions of the second law of thermodynamics proposed so far required an infinite bath and ideal energy storage in order to be tight. Here, Łobejko loosens these requirements, proving a tight upper bound on the average work that can be extracted in a quantum scenario.

## Introduction

Quantum thermodynamics is an emerging theory with the primary goal to generalize the Laws of Thermodynamics, valid in a macroscopic domain, to the energetic description of individual quantum systems. Alongside many other approaches, recently, quantum thermodynamics has been formulated as a general unitary dynamics and a resource theory of non-equilibrium quantum states^1–9^. The fundamental question to answer in this framework is: how much work can be extracted providing a particular resource?

To answer this question, in the first place, one should define what is really the work in a quantum domain, since different definitions vary from one to another framework and regimes of interests^2,3,5,6,10–12,13–20^. The lack of consensus in this field is mainly due to the presence of coherences in quantum states^7,21–26^ and the appearance of work fluctuations^27–30^. For autonomous thermal machines, where the work reservoir is explicit, one of the most promising concepts is the translationally-invariant energy storage (with dynamics equivalent to the physical weight), where a change of its average energy corresponds to the work^5^.

It was shown that the work reservoir given by the weight is consistent with fluctuations theorems^31–33^, it can be used to derive the Third Law of Thermodynamics^34^ or to an analysis of the optimal performance of heat engines^35,36^. In particular, according to the research introducing the weight idea^5^, Skrzypczyk et al. proved that the optimal extracted work *W* from a quantum state ${\hat{\rho }}_{S}$, in contact with a thermal reservoir at temperature *T* = *β*^−1^, is bounded by the difference of its non-equilibrium free energy:

1 $$W\le F({\hat{\rho }}_{S})-F({\hat{\tau }}_{S})=TS({\hat{\rho }}_{S}| | {\hat{\tau }}_{S}),$$

where $F(\hat{\rho })=E(\hat{\rho })-TS(\hat{\rho })$ and $E(\hat{\rho })$ is the average energy, $S(\hat{\rho })$ is the von Neumann entropy and ${\hat{\tau }}_{S}={{\mathcal{Z}}}_{S}^{-1}{e}^{-\beta {\hat{H}}_{S}}$ is a Gibbs state according to the free Hamiltonian ${\hat{H}}_{S}$ with partition function ${{\mathcal{Z}}}_{S}$. $S(\hat{\rho }| | \hat{\eta })={\rm{Tr}}[\hat{\rho }({\mathrm{log}}\,\hat{\rho} -{\mathrm{log}}\,{\hat{\eta}} )]$ is the quantum relative entropy.

Inequality (1) encapsulates the quantum form of the Second Law of Thermodynamics, which especially restricts all possible micro engines to operate below the universal Carnot efficiency. However, as presented by the authors, the optimal work ${W}_{\text{max}}=F({\hat{\rho }}_{S})-F({\hat{\tau }}_{S})$ is only attainable under two strong conditions: (i) the requirement of an infinite heat bath and (ii) the average energy conservation. The first assumption is required to split the protocol into an infinite number of steps, such that the optimal work can be extracted in a quasi-reversible process. However, it is seen that the saturation of the Second Law is never possible for the physical (i.e., finite) heat baths. On the other hand, the second assumption is imposed in order to make possible the full work extraction from coherences, but in this approach, the First Law is not independent of the initial state. On the contrary, imposing the strict form of the energy-conservation once again (in general) makes the Second Law not tight^22^. In other words, as long as the inequality (1) provides the universal upper bound for the work extraction, it does not answer the question of when it can be saturated, which requires additional information about microscopic details of the heat bath and state of the work reservoir.

The work extraction process with the work reservoir given by the weight was recently studied (within the context of heat engines) for isolated systems^36^, where it was proved that the work flow is limited by the ergotropy of the effective state, the so-called control-marginal state. On the other hand, a concept of the ergotropy as the maximal extractable work naturally arises from the cyclic non-autonomous protocols of closed quantum systems (with implicit work reservoirs)^37^, which is an intensively studied area of so-called ‘quantum batteries’^38–49^. One of the most important concepts coming from the work extraction via the unitary channels is a generalization of the equilibrium state (the minimal energy state with fixed entropy) to the larger class of the passive states (the minimal energy states with fixed spectrum of a density operator).

In this article, we use the above concepts and study the work extraction process from quantum systems in contact with (finite-size) heat baths, which establish the tight Second Law inequality. In particular, we reveal that the control-marginal state solely quantifies the locked energy in coherences, and the free energy difference between the corresponding passive state and the equilibrium state is equal to the locked energy in a finite-size thermal reservoir.

## Results

### Quantum weight and work extraction process

We consider the work extraction process as the unitary evolution ${\hat{\rho }}_{SW}\otimes {\hat{\tau }}_{B}\to \hat{U}{\hat{\rho }}_{SW}\otimes {\hat{\tau }}_{B}{\hat{U}}^{\dagger }$, where ${\hat{\rho }}_{SW}$ is the arbitrary joint state of the system and the weight, and ${\hat{\tau }}_{B}={{\mathcal{Z}}}_{B}^{-1}{e}^{-\beta {\hat{H}}_{B}}$ is the Gibbs state of the bath (with the partition function ${{\mathcal{Z}}}_{B}$). Next, we impose strict energy conservation in the form:

2 $$[\hat{U},{\hat{H}}_{S}+{\hat{H}}_{B}+{\hat{H}}_{W}]=0,$$

where ${\hat{H}}_{S},{\hat{H}}_{B}$ and ${\hat{H}}_{W}$ are corresponding free Hamiltonians of the subsystems. The symmetry ensures that the average energy is conserved for the arbitrary initial state before and after a protocol. Notice that tracing out the bath, the channel ${{\Lambda }}[{\hat{\rho }}_{SW}]={{\rm{Tr}}}_{B}[\hat{U}{\hat{\rho }}_{SW}\otimes {\hat{\tau }}_{B}{\hat{U}}^{\dagger }]$ is the thermal operation^2^.

The second most important ingredient for the framework of thermodynamics (after the energy conservation) is a proper definition of work. Here, for an autonomous system, it is done by a definition of the work reservoir. Specifically, we consider the weight model with the Hamiltonian ${\hat{H}}_{W}=\int d\epsilon \,\epsilon \left|\epsilon \right\rangle {\left\langle \epsilon \right|}_{W}$ (where ${\left|\epsilon \right\rangle }_{W}$ is the energy eigenstate), and we postulate the translational symmetry in the form^31,34^:

3 $$[\hat{U},{\hat{{{\Delta }}}}_{W}]=0,$$

where ${\hat{{{\Delta }}}}_{W}$ is the generator of shifts in the energy spectrum of the weight, i.e., ${\hat{{{\Gamma }}}}_{\varepsilon }={e}^{-i{\hat{{{\Delta }}}}_{W}\varepsilon }$ such that ${\hat{{{\Gamma }}}}_{\varepsilon }^{\dagger }{\hat{H}}_{W}{\hat{{{\Gamma }}}}_{\varepsilon }={\hat{H}}_{W}+\varepsilon$ for arbitrary real *ϵ* (for more details, see Supplementary Notes 1 and 2). Finally, work is defined as a change of the average energy of the weight, namely

4 $$W:={\rm{Tr}}[{\hat{H}}_{W}(\hat{U}{\hat{\rho }}_{SW}\otimes {\hat{\tau }}_{B}{\hat{U}}^{\dagger }-{\hat{\rho }}_{SW}\otimes {\hat{\tau }}_{B})].$$

To interpret the physical meaning of the weight model (and especially the translational symmetry), let us consider the classical definition of work given by a displacement of the system *δx* times the force *F* acting on it, i.e., *δW* = *Fδx*. According to this analogy, one should notice that the Hamiltonian ${\hat{H}}_{W}$ can be mapped into the form ${\hat{H}}_{W}=F\hat{x}$, where $\hat{x}$ is the position operator. In this sense, the energy is stored as potential energy within an external homogeneous field (e.g., gravitational field). However, the most important thing that comes from this analogy is the physical meaning of the symmetry given by Eq. (3), which simply reveals the isotropy of space, i.e., in the homogeneous field, the protocol should not depend on the absolute position of the system.

In this sense, the Hamiltonian ${\hat{H}}_{W}$ obeying condition (3) can be seen as the ‘quantized’ version of the classical work definition. Notice that here we replace the scalar with the operator, such that the expected value also involves the quantum state ${\hat{\rho }}_{W}$. As a consequence, the definition of the (‘quantized’) work is enriched by purely quantum effects, like work extraction from coherences (present in the state ${\hat{\rho }}_{W}$).

### Tight Second Law

We reveal that for arbitrary protocol ${\hat{\rho }}_{SW}\otimes {\hat{\tau }}_{B}\to \hat{U}{\hat{\rho }}_{SW}\otimes {\hat{\tau }}_{B}{\hat{U}}^{\dagger }$, where $\hat{U}$ is the energy-conserving and translationally-invariant unitary (Eqs. (2) and (3)), the tight Second Law can be written in the form:

5 $$W\le R({\hat{\sigma }}_{S}\otimes {\hat{\tau }}_{B}),$$

where $R(\hat{\rho })$ is the ergotropy of the state $\hat{\rho }$^37^:

6 $$R(\hat{\rho }):=E(\hat{\rho })-E({\mathcal{P}}[\hat{\rho }]),\,\,{\mathcal{P}}[\hat{\rho }]:=\arg \ {\min }_{\hat{U}-\text{unitary}}E(\hat{U}\hat{\rho }{\hat{U}}^{\dagger }),$$

i.e., the maximal energy extracted from an arbitrary unitary channel $\hat{\rho }\to \hat{U}\hat{\rho }{\hat{U}}^{\dagger }$ with fixed Hamiltonian $\hat{H}$, where ${\mathcal{P}}[\hat{\rho }]$ is the passive state^50^ (i.e., the minimal energy state with fixed spectrum). According to the quantity $R({\hat{\sigma }}_{S}\otimes {\hat{\tau }}_{B})$ the constant Hamiltonian is equal to ${\hat{H}}_{S}+{\hat{H}}_{B}$, and ${\hat{\sigma }}_{S}$ is the so-called control-marginal state^36^:

7 $${\hat{\sigma }}_{S}:={{\rm{Tr}}}_{W}[{e}^{-i{\hat{H}}_{S}\otimes {\hat{{{\Delta }}}}_{W}}{\hat{\rho }}_{SW}{e}^{i{\hat{H}}_{S}\otimes {\hat{{{\Delta }}}}_{W}}].$$

From now to the end of this article, we will consider only product states, i.e., ${\hat{\rho }}_{SW}={\hat{\rho }}_{S}\otimes {\hat{\rho }}_{W}$, such that the control-marginal operator is given as a mixture of unitaries:

8 $${\hat{\sigma }}_{S}=\int dt\,p(t)\,{\hat{U}}_{t}{\hat{\rho }}_{S}{\hat{U}}_{t}^{\dagger },$$

where $p(t)={\rm{Tr}}[{\hat{\rho }}_{W}\left|t\right\rangle {\left\langle t\right|}_{W}]$, ${\hat{U}}_{t}={e}^{-i{\hat{H}}_{S}t}$ and ${\left|t\right\rangle }_{W}$ is an eigenstate of the shift generator ${\hat{{{\Delta }}}}_{W}$, i.e., the canonically conjugate ‘time state’ with respect to the energy eigenvectors ${\left|\varepsilon \right\rangle }_{W}$, such that ${\left|t\right\rangle }_{W}=\int d\epsilon \,{e}^{i\epsilon t}{\left|\epsilon \right\rangle }_{W}$. We refer the reader to Supplementary Note 3 for the derivation of Eq. (8) and Supplementary Note 4 for the proof of Eq. (5).

Inequality (5) presents that the optimal work done on a weight via energy-conserving unitary dynamics is equal to the ergotropy of the composite state ${\hat{\sigma }}_{S}\otimes {\hat{\tau }}_{B}$. Furthermore, we reveal that it can be expressed in the form:

9 $$R({\hat{\sigma }}_{S}\otimes {\hat{\tau }}_{B})=TS({\hat{\rho }}_{S}| | {\hat{\tau }}_{S})-{{{\Delta }}}_{B}({\hat{\rho }}_{S},{\hat{\tau }}_{B})-{{{\Delta }}}_{C}({\hat{\rho }}_{S},{\hat{\rho }}_{W},{\hat{\tau }}_{B}),$$

where ${{{\Delta }}}_{B}({\hat{\rho }}_{S},{\hat{\tau }}_{B})$ and ${{{\Delta }}}_{C}({\hat{\rho }}_{S},{\hat{\rho }}_{W},{\hat{\tau }}_{B})$ are non-negative corrections to the ultimate thermodynamic bound given by Eq. (1). The former has a non-vanishing value for the finite-size heat baths, and the latter is solely connected with the presence of the locked energy in coherences. These two quantities, the main object of this research, are defined and discussed in detail in the following subsections.

### Work extraction via contact with a finite-size heat bath

Let us start with an analysis of the work extraction process from the system in contact with a finite-size heat reservoir. One should observe that the optimal work $R({\hat{\sigma }}_{S}\otimes {\hat{\tau }}_{B})$ depends explicitly on the heat bath equilibrium state ${\hat{\tau }}_{B}$, which is defined both by the temperature *T* and the Hamiltonian ${\hat{H}}_{B}$. On the contrary, the only information coming from the environment included in the Second Law given by Eq. (1), formulated solely in terms of the free energy, is the temperature *T*. This ignorance of microscopic details of the thermal reservoir as a consequence makes the inequality in general not tight.

Now, we would like to state a general relation between ergotropy and free energy for quantum systems coupled to the heat bath. We show that for arbitrary quantum state ${\hat{\rho }}_{S}$ with Hamiltonian ${\hat{H}}_{S}$ and arbitrary Gibbs state ${\hat{\tau }}_{B}$ with Hamiltonian ${\hat{H}}_{B}$ the ergotropy of a composite system can be expressed as:

10 $$R({\hat{\rho }}_{S}\otimes {\hat{\tau }}_{B})	=E({\hat{\rho }}_{S}\otimes {\hat{\tau }}_{B})-E({\mathcal{P}}[{\hat{\rho }}_{S}\otimes {\hat{\tau }}_{B}])=F({\hat{\rho }}_{S}\otimes {\hat{\tau }}_{B})-F({\mathcal{P}}[{\hat{\rho }}_{S}\otimes {\hat{\tau }}_{B}])\\ 	=F({\hat{\rho }}_{S}\otimes {\hat{\tau }}_{B})-F({\hat{\tau }}_{S}\otimes {\hat{\tau }}_{B})-F({\mathcal{P}}[{\hat{\rho }}_{S}\otimes {\hat{\tau }}_{B}])+F({\hat{\tau }}_{S}\otimes {\hat{\tau }}_{B})\\ 	=T\left[\right.S({\hat{\rho }}_{S}| | {\hat{\tau }}_{S})-S({\mathcal{P}}[{\hat{\rho }}_{S}\otimes {\hat{\tau }}_{B}]| | {\hat{\tau }}_{S}\otimes {\hat{\tau }}_{B})\left]\right.,$$

where ${\hat{\tau }}_{S}$ is the Gibbs state according to the Hamiltonian ${\hat{H}}_{S}$, and both the Gibbs states likewise the free energy are defined with respect to the same and arbitrary temperature *T*. In the first line, we use the fact that $S({\hat{\rho }}_{S}\otimes {\hat{\tau }}_{B})=S({\mathcal{P}}[{\hat{\rho }}_{S}\otimes {\hat{\tau }}_{B}])$. Next, according to Eq. (9), we define a quantity that describes the inability of extracting work from a given heat bath in the state ${\hat{\tau }}_{B}$, namely

11 $${{{\Delta }}}_{B}({\hat{\rho }}_{S},{\hat{\tau }}_{B}) 	:=TS({\hat{\rho }}_{S}| | {\hat{\tau }}_{S})-R({\hat{\rho }}_{S}\otimes {\hat{\tau }}_{B})\\ 	=TS\left(\right.{\mathcal{P}}[{\hat{\rho }}_{S}\otimes {\hat{\tau }}_{B}]| | {\hat{\tau }}_{S}\otimes {\hat{\tau }}_{B}\left)\right.\ge 0.$$

It provides a measure of how the corresponding thermal reservoir is able to extract free energy from the non-equilibrium quantum state, and we call it the locked energy in a finite-size bath. The expression (11) shows that this bounded energy is proportional to the relative entropy between the global passive state and the corresponding equilibrium state, which provides an interesting measure of the thermal reservoir’s effectiveness. Moreover, the non-negativity of this quantity directly proves the Second Law, such that the maximal extracted work (ergotropy) never exceeds the non-equilibrium free energy, i.e., $R({\hat{\rho }}_{S}\otimes {\hat{\tau }}_{B})\le TS({\hat{\rho }}_{S}| | {\hat{\tau }}_{S})$ (see Supplementary Note 5 for extended analysis and alternative derivation).

Finally, let us examine how the locked energy (11) behaves with respect to the growing size of the bath. We consider a thermal reservoir composed of *N* subsystems (e.g., qubits, see subsection Example) such that its total (Gibbs) state is denoted by ${\hat{\tau }}_{B}^{(N)}$. Next, let us note that $R({\hat{\rho }}_{S}\otimes {\hat{\tau }}_{B}^{(N+1)})\ge R({\hat{\rho }}_{S}\otimes {\hat{\tau }}_{B}^{(N)})$, i.e., the ergotropy cannot decrease with respect to *N* since the optimization (6) for a smaller heat bath is done over the subset of all possible unitaries acting on the greater Hilbert space. As a consequence, we prove that the locked energy is always non-increasing with the growing size of the thermal reservoir, namely ${{{\Delta }}}_{B}({\hat{\rho }}_{S},{\hat{\tau }}_{B}^{(N+1)})\le {{{\Delta }}}_{B}({\hat{\rho }}_{S},{\hat{\tau }}_{B}^{(N)})$. Furthermore, according to the result of Skrzypczyk et al.^5^, for some specific heat baths in the thermodynamic limit, it is possible to achieve that for the arbitrary state of the system, the locked energy vanishes, i.e.,

12 $${\forall }_{\hat{\rho }_{S}}\ {\mathrm{lim}\,}_{N\to \infty }{{{\Delta }}}_{B}\Big({\hat{\rho }}_{S},{\hat{\tau }}_{B}^{(N)}\Big)=0.$$

This proves that the bound $R({\hat{\rho }}_{S}\otimes {\hat{\tau }}_{B})=TS({\hat{\rho }}_{S}| | {\hat{\tau }}_{S})$ is attainable for the generic macroscopic thermal reservoirs, which in some sense shows that the ergotropy $R({\hat{\rho }}_{S}\otimes {\hat{\tau }}_{B})$ is a generalization of the free energy for the finite-size heat baths.

### Work extraction from coherences

Next, we turn to the second (fully quantum) contribution that decreases the optimal work in Eq. (9). One of the most interesting features of quantum thermodynamics is a possibility of extracting work from the quantum coherence. Notice, however, that the symmetry given by Eq. (2) provides not only that the energy flow is conserved, but it also imposes the additional constraints on the manipulation of the off-diagonal elements (between different energy eigenstates). In general, this leads to the phenomenon known as ‘work-locking’^22^, i.e., the inability of the free energy (or ergotropy) extraction contributed from the quantum coherences.

To understand this phenomenon for the energy storage given by the quantum weight, firstly, one should notice that the optimal work $R({\hat{\sigma }}_{S}\otimes {\hat{\tau }}_{B})$ depends implicitly on the state of the work reservoir ${\hat{\rho }}_{W}$ through the control-marginal state ${\hat{\sigma }}_{S}$. We point out, however, that the channel (8) only affects the off-diagonal elements of the density matrix ${\hat{\rho }}_{S}$, i.e., if $[{\hat{\rho }}_{S},{\hat{H}}_{S}]=0$ then ${\hat{\sigma }}_{S}={\hat{\rho }}_{S}$. From this follows that for quasi-classical diagonal states, the work extraction protocol is independent of the weight state at all. Nevertheless, if we consider a coherent state ${\hat{\rho }}_{S}$, it is no longer true, i.e., in general ${\hat{\sigma }}_{S}\ne {\hat{\rho }}_{S}$, and the optimal value of work indirectly depends on the state of the weight (and especially of its amount of coherences). In order to quantify how much of the energy is bounded, we define the locked energy in coherences (see Eq. (9)):

13 $${{{\Delta }}}_{C}({\hat{\rho }}_{S},{\hat{\rho }}_{W},{\hat{\tau }}_{B}):=R({\hat{\rho }}_{S}\otimes {\hat{\tau }}_{B})-R({\hat{\sigma }}_{S}\otimes {\hat{\tau }}_{B})\ge 0,$$

i.e., a quantum thermodynamic resource that cannot be extracted as work via a state of the weight ${\hat{\rho }}_{W}$. The non-negativity of this quantity is ensured by a definition of the channel (8), given as a mixture of unitaries, and proved in Supplementary Note 6.

In this way, we can introduce a concept of the ideal weight, i.e., an energy storage system that is able to the full work extraction from coherences with Δ_*C*_ = 0. In particular, this is the case if the state of the weight tends to the time state, i.e., ${\hat{\rho }}_{W}\to \left|t\right\rangle {\left\langle t\right|}_{W}$, such that we have ${\hat{\sigma }}_{S}\to {\hat{U}}_{t}{\hat{\rho }}_{S}{\hat{U}}_{t}^{\dagger }$ and Δ_*C*_ → 0. The time state of the weight is an extreme and idealized example of the system with an ‘infinite amount of coherence’ (see e.g.,^3,22^), and in this sense, it can perform a unitary transformation on the subsystem and achieve the optimal work extraction. In the opposite limit, where the energy storage tends to the energy eigenstate, i.e., ${\hat{\rho }}_{W}\to \left|\varepsilon \right\rangle {\left\langle \varepsilon \right|}_{W}$, the control-marginal state loses all of the coherences, such that ${\hat{\sigma }}_{S}\to {\mathcal{D}}[{\hat{\rho }}_{S}]$ (where ${\mathcal{D}}[\cdot ]$ is a dephasing channel in the energy basis), and hence Δ_*C*_ is maximal. The fact that ${\hat{\sigma }}_{S}={\mathcal{D}}[{\hat{\rho }}_{S}]$ for incoherent states of the work reservoirs was previously observed^22^, where the authors discuss the work extraction from coherences via the additional ancillary system, a source of coherence, acting as a catalyst. Here, on the contrary, we allow to use the coherent states of the weight (i.e., a fully quantum energy storage) and reveal how this can ‘unlock’ the extracted work, where via the control-marginal state, we are able to quantify all the intermediate cases.

Finally, we would like to derive the thermodynamic limit of the locked energy Δ_*C*_. Firstly, let us rewritten Eq. (13) in the form:

14 $${{{\Delta }}}_{C}({\hat{\rho }}_{S},{\hat{\rho }}_{W},{\hat{\tau }}_{B})	=T[S({\hat{\rho }}_{S}| | {\hat{\tau }}_{S})-S({\hat{\sigma }}_{S}| | {\hat{\tau }}_{S})]-{{{\Delta }}}_{B}({\hat{\rho }}_{S},{\hat{\tau }}_{B})+{{{\Delta }}}_{B}({\hat{\sigma }}_{S},{\hat{\tau }}_{B})\\ 	=F({\hat{\rho }}_{S})-F({\hat{\sigma }}_{S})-{{{\Delta }}}_{B}({\hat{\rho }}_{S},{\hat{\tau }}_{B})+{{{\Delta }}}_{B}({\hat{\sigma }}_{S},{\hat{\tau }}_{B})\\ 	=T\left[S({\hat{\sigma }}_{S})-S({\hat{\rho }}_{S})\right]-{{{\Delta }}}_{B}({\hat{\rho }}_{S},{\hat{\tau }}_{B})+{{{\Delta }}}_{B}({\hat{\sigma }}_{S},{\hat{\tau }}_{B}),$$

where we have used Eq. (11), and the last equality follows from the fact that $E({\hat{\rho }}_{S})=E({\hat{\sigma }}_{S})$. Next, if for the heat bath ${\hat{\tau }}_{B}^{(N)}$ holds Eq. (12), then

15 $${\mathrm{lim}\,}_{N\to \infty }{{{\Delta }}}_{C}({\hat{\rho }}_{S},{\hat{\rho }}_{W},{\hat{\tau }}_{B}^{(N)})=T\left[S({\hat{\sigma }}_{S})-S({\hat{\rho }}_{S})\right].$$

Equation (15) is an interesting formula showing that the thermodynamic limit of the locked energy in coherences is equal to the difference of entropy between the control-marginal state ${\hat{\sigma }}_{S}$ and state ${\hat{\rho }}_{S}$, multiplied by the bath temperature *T*. This characterizes the work extraction process from quantum coherences in the presence of the macroscopic heat bath. However, we would like to emphasize that this quantity is not the upper bound of the locked energy Δ_*C*_ (as the free energy is for the optimal work $R({\hat{\sigma }}_{S}\otimes {\hat{\tau }}_{B})$), but rather it is the thermodynamic limit. In the next paragraph, we provide a numerical simulation of a particular example where the locked energy for the finite-size bath can be bigger than the value given by Eq. (15), and moreover, it can be even non-monotonic with respect to the growing size of the thermal reservoir.

### Example

Let us now consider a particular example to illustrate how the finite-size bath and state of the weight affect the work extraction process. We would like to concentrate on a system *S* given by the qubit in a coherent ‘plus state’, i.e., ${\hat{\rho }}_{S}=\left|+\right\rangle {\left\langle +\right|}_{S}$, where ${\left|+\right\rangle }_{S}=\frac{1}{\sqrt{2}}({\left|0\right\rangle }_{S}+{\left|1\right\rangle }_{S})$, and with Hamiltonian ${\hat{H}}_{S}=\omega \left|1\right\rangle {\left\langle 1\right|}_{S}$. Next, as a model of a bath, we take a collection of qubits with different energy gaps, namely the bath Hamiltonian is given by

16 $${\hat{H}}_{B}^{(N)}=\mathop{\bigotimes }\limits_{k=1}^{N}{\omega }_{k}\left|{1}_{k}\right\rangle {\left\langle {1}_{k}\right|}_{B},$$

where ${\omega }_{k}=T\mathrm{log}\,[\frac{1-k\delta }{k\delta }]$ and $\delta ={{\mathcal{Z}}}_{S}^{-1}{e}^{-\beta \omega }/N$. The choice of the heat bath is dictated by its property that in the limit of infinite number of qubits the Eq. (12) is satisfied^5^. Finally, we take the weight in a pure state given by a Gaussian superposition of energy states, i.e., ${\hat{\rho }}_{W}=\left|\psi \right\rangle {\left\langle \psi \right|}_{W}$ such that

17 $${\left|\psi \right\rangle }_{W}={(2\pi {\sigma }^{2})}^{-1/4}\int d\varepsilon \,{e}^{-\frac{{\epsilon }^{2}}{4{\sigma }^{2}}}{\left|\varepsilon \right\rangle }_{W},$$

where the standard deviation σ solely parameterizes the vector.

Within this model, the optimal work is equal to ${W}_{\text{max}}=R\left(\left|+\right\rangle {\left\langle +\right|}_{S}\otimes {\hat{\tau }}_{B}^{(N)}\right)$, where the state ${\hat{\tau }}_{B}^{(N)}$ corresponds to the bath composed of *N* qubits (16). We numerically calculate the locked energy in a finite-size bath, which is given by

18 $${{{\Delta }}}_{B}({\hat{\rho }}_{S},{\hat{\tau }}_{B}^{(N)})=\frac{\omega }{2}+T{\mathrm{log}}\,[1+{\mathrm{e}}^{-\beta \omega }]-{\mathrm{R}}\left(\left|+\right\rangle {\left\langle +\right|}_{\mathrm{S}}\otimes {\hat{\tau }}_{\mathrm{B}}^{(\mathrm N)}\right),$$

and the locked energy in coherences:

19 $${{{\Delta }}}_{C}({\hat{\rho }}_{S},{\hat{\rho }}_{W},{\hat{\tau }}_{B}^{(N)})=R\left(\left|+\right\rangle {\left\langle +\right|}_{S}\otimes {\hat{\tau }}_{B}^{(N)}\right)-R\left({\hat{\xi }}_{S}\otimes {\hat{\tau }}_{B}^{(N)}\right)-\frac{\omega \gamma }{2},$$

where $$\gamma =\exp [-\frac{{\omega }^{2}}{8{\sigma }^{2}}]$$ and

20 $${\hat{\xi }}_{S}=\frac{1}{2}(1+\gamma )\left|0\right\rangle {\left\langle 0\right|}_{S}+\frac{1}{2}(1-\gamma )\left|1\right\rangle {\left\langle 1\right|}_{S}.$$

**Fig. 1:**
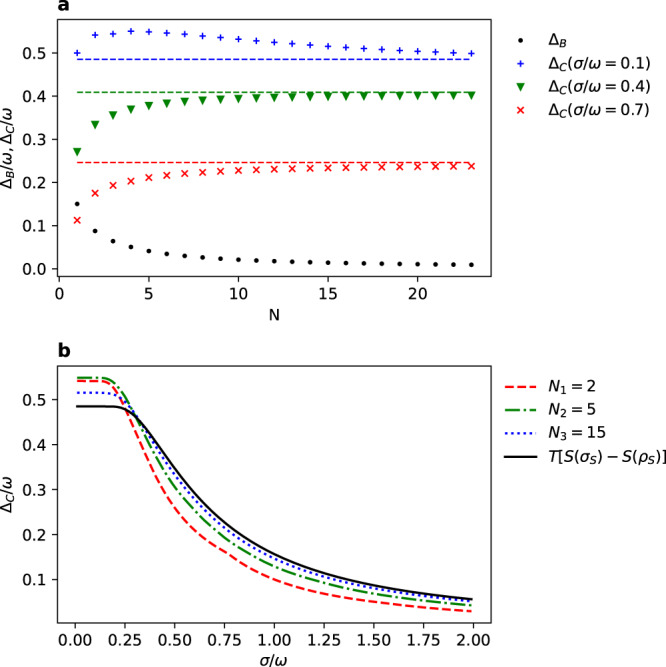
Locked energy for a qubit in the state ${\hat{\rho }}_{S}=\left|+\right\rangle {\left\langle +\right|}_{S}$ and with the energy gap *ω*. **a** Graph presents how the locked energy in a finite-size bath ${{{\Delta }}}_{B}({\hat{\rho }}_{S},{\hat{\tau }}_{B}^{(N)})$ and locked energy in coherences ${{{\Delta }}}_{C}({\hat{\rho }}_{S},{\hat{\rho }}_{W},{\hat{\tau }}_{B}^{(N)})$ depends on the number of qubits *N* in the heat bath (16), for different values of the scaled standard deviation *σ*/*ω* of the Gaussian state of the weight (17). Horizontal lines correspond to the thermodynamic limit (*N* → *∞*) of the locked energy in coherences expressed by the entropy difference: $T[S({\hat{\sigma }}_{S})-S({\hat{\rho }}_{S})]$. **b** The vanishing of the locked energy in coherences for a different size of the heat bath *N* with respect to the parameter *σ*/*ω*. The temperature of the bath is equal to *T* = 0.7*ω*.

The results are presented in Fig. 1. It is observed that the locked energy in a finite-size bath ${{{\Delta }}}_{B}({\hat{\rho }}_{S},{\hat{\tau }}_{B}^{(N)})$ vanishes for a large number of qubits *N*, providing that the ergotropy $R({\hat{\rho }}_{S}\otimes {\hat{\tau }}_{B})\to TS({\hat{\rho }}_{S}| | {\hat{\tau }}_{S})$. Similarly, we analyze the locked energy in coherences ${{{\Delta }}}_{C}({\hat{\rho }}_{S},{\hat{\rho }}_{W},{\hat{\tau }}_{B}^{(N)})$, where for the Gaussian model of the weight it depends only via a single parameter *σ*/*ω*, i.e., a ratio between a standard deviation of the work reservoir wave packet *σ* and the energy gap of a qubit *ω*. In Fig. 1a, the computed values are compared to the derived thermodynamic limit given by $T[S({\hat{\sigma }}_{S})-S({\hat{\rho }}_{S})]$(15). An intriguing observation is that as long as the locked energy in a finite-size bath decreases monotonically (as it was proven previously), the locked energy in coherences (for some values of *σ*/*ω*) is not necessarily monotonic function.

Further, in Fig. 1b, we present how quickly the locked energy in coherences vanishes with increasing value of the ratio *σ*/*ω*. Two interesting features are observed here. First, for high values of *σ*/*ω*, the locked energy is increasing with the size of the bath *N*, however, this order is changed for low values and becomes non-monotonic. Secondly, for low values, we observe a plateau, i.e., the locked energy almost stays constant with the growing width of the wave packet. Notice that in the limit *σ* → 0 state of the weight tends to the energy state with the maximal locked energy, and in the limit *σ* → *∞* it tends to the time state for which the locked energy vanishes.

## Discussion

The ergotropy is a proper resource regarding the work extraction process if an explicit energy storage is given by the translationally-invariant weight. This quantity on its own can be defined as the optimal work extracted from closed systems driven by the time-dependent and cyclic Hamiltonians, which proves an important connection between those two frameworks. On the other hand, we reveal that there is no full equivalence since models with implicit energy storage do not involve the concept of the locked energy in coherences, i.e., the off-diagonal part that contributes to ergotropy (or free energy) but cannot be extracted as a work. Indeed, one of the main differences between classical and quantum thermodynamics is that quantum systems are able to perform the work via coherences. However, it is only possible if the work reservoir has coherences as well, and the locked energy naturally emerges if we treat it explicitly. In other words, for the quantum process of work extraction, the weight must be the energy reservoir and the reservoir of coherences likewise. We provide a quantitative definition of the bounded energy in coherences in terms of the effective control-marginal state. In particular, it reveals the condition for the ideal work reservoir, i.e., the energy storage that is able to the full work extraction from coherences (which is really the case in the non-autonomous approach).

Furthermore, we analyze the ergotropy of the non-equilibrium quantum system in contact with an arbitrary finite-size heat bath. In the light of the resource theory, such a Gibbs state of the bath is treated as costless, i.e., it can be for free attached to, and discarded from the system. Due to the non-additivity of the ergotropy, the heat bath activates the non-equilibrium state of the system, and consequently both of them form the entire thermodynamic resource (given by the total ergotropy). This can be simply interpreted as a maximal work that can be extracted from such a quantum state. Moreover, the second most important result of this work is establishing the general relation between the ergotropy and free energy for systems coupled to the heat bath, which provides a bridge between microscopic and macroscopic thermodynamics. We show that the total ergotropy of the quantum system and finite-size heat bath never exceeds the non-equilibrium free energy, whereas the difference is proportional to the relative entropy between the global passive state and the corresponding equilibrium state. Furthermore, this kind of locked energy (due to the finiteness of the thermal environment) vanishes in the thermodynamic limit for the generic macroscopic heat baths. This suggests that the ergotropy of the composite state of the system and thermal reservoir can be interpreted as the generalization of the free energy for the finite-size heat bath. Moreover, the relation between the ergotropy and free energy leads us to the thermodynamic limit of the locked energy in coherences. The limit provides an interesting formula, expressed in terms of the von Neumann entropy, that from one side is fully quantum since it refers to the extraction of work from coherences (i.e., requires the coherent state of the system and the energy storage likewise), but on the other side involves the classical notion of the macroscopic heat bath.

Finally, we would like to emphasize that the presented here model can be further slightly modified. In particular, the optimal work is still equal to the ergotropy $R({\hat{\sigma }}_{S}\otimes {\hat{\tau }}_{B})$ if we consider the correlated states ${\hat{\rho }}_{SW}$ with the control-marginal state equal to (7). Furthermore, the energy-conservation relation (2) can be generalized to $[\hat{U},{\hat{H}}_{SB}+{\hat{H}}_{W}]=0$, where one can include a non-vanishing interaction term between the system and the bath before and after the protocol. However, in this case, the optimal work is given by $R({\hat{\sigma }}_{SB})$, where one should take the total Hamiltonian ${\hat{H}}_{SB}$ rather than ${\hat{H}}_{S}+{\hat{H}}_{B}$, and we have a joint control-marginal state ${\hat{\sigma }}_{SB}$ (calculated from Eq. (7) with the Hamiltonian ${\hat{H}}_{SB}$ and the total state ${\hat{\rho }}_{SBW}$). The motivation to consider here the simplest formulation (i.e., the product states and energy-conservation of free Hamiltonians) ensures that both ${{{\Delta }}}_{C}({\hat{\rho }}_{S},{\hat{\rho }}_{W},{\hat{\tau }}_{B})$ and ${{{\Delta }}}_{B}({\hat{\rho }}_{S},{\hat{\tau }}_{B})$ are non-negative. The interesting question of how those quantities would be changed if we relax those assumptions we let open for future studies.

## Supplementary information

Supplementary Information

## Data Availability

The data that support the findings of this study are available from the author upon reasonable request.
