# Supplementary material for: The tight Second Law inequality for coherent quantum systems and finite-size heat baths
Source: Nat Commun. 2021 Feb 10;12:918. doi: 10.1038/s41467-021-21140-4 (PMC7876128; doi:10.1038/s41467-021-21140-4)
Supplement: Supplementary file 1 — Supplementary Information [file 41467_2021_21140_MOESM1_ESM.pdf]

Supplementary Information

The tight Second Law inequality for coherent quantum systems and finite-size heat baths, M. Łobejko

Supplementary Note 0 – Average energy, passive energy and ergotropy

Let us consider a system $\hat{\rho}$ with free Hamiltonian $\hat{H}$. We define the following quantities:

$$E(\hat{\rho}) = \mathrm{Tr}\big[\hat{H}\hat{\rho}\big], \; P(\hat{\rho}) = \min_{\hat{V}} \mathrm{Tr}\big[\hat{V}^\dagger \hat{H} \hat{V} \hat{\rho}\big], \; R(\hat{\rho}) = E(\hat{\rho}) - P(\hat{\rho}), \tag{1}$$

i.e. average energy $E(\hat{\rho})$, passive energy $P(\hat{\rho})$ and ergotropy $R(\hat{\rho})$[1], where $\hat{V}$ is arbitrary unitary acting on the system Hilbert space.

Supplementary Note 1 – Energy and time states of the weight

We assume that the energy storage is given by the weight. In particular, this implies that its energy spectrum is continuous, namely

$$\hat{H}_W = \int d\varepsilon \; \varepsilon \, |\varepsilon\rangle\langle\varepsilon|_W \, . \tag{2}$$

We further define time states $|t\rangle_W$ as the conjugate states with respect to the energy vectors $|\varepsilon\rangle_W$, i.e. transition from one to another basis is given by the Fourier transform:

$$|t\rangle_W = \int d\varepsilon \; e^{i\varepsilon t} \, |\varepsilon\rangle_W \, . \tag{3}$$

Next, we consider a shift operator which is defined by its action on energy eigenstates: $\hat{\Gamma}_\delta \, |\varepsilon\rangle_W = |\varepsilon + \delta\rangle_W$. From this relation it follows that:

$$\hat{\Gamma}_\delta \, |t\rangle_W = \int d\varepsilon \; e^{i\varepsilon t} \, \hat{\Gamma}_\delta \, |\varepsilon\rangle_W = \int d\varepsilon \; e^{i\varepsilon t} \, |\varepsilon + \delta\rangle_W = e^{-i\delta t} \int d\varepsilon \; e^{i\varepsilon t} \, |\varepsilon\rangle_W = e^{-i\delta t} \, |t\rangle_W \, , \tag{4}$$

i.e. time states are eigenstates of the shift operator. According to this definition one can further define an operator:

$$\hat{\Delta}_W = \int dt \; t \; |t\rangle\langle t|_W \, , \tag{5}$$

which is the generator of shifts, i.e. $\hat{\Gamma}_\delta = e^{-i\hat{\Delta}_W \delta}$. This obeys the canonical commutation relation with the Hamiltonian $\hat{H}_W$ in the form: $[\hat{H}_W, \hat{\Delta}_W] = i$.

Supplementary Note 2 – Energy-conserving and translationally-invariant unitary

We consider a quantum system coupled to a heat bath, prepared in a Gibbs state, and the energy storage given by the weight. Then, we investigate a unitary protocol $\hat{\rho}_{SW} \otimes \hat{\tau}_B \to \hat{U} \hat{\rho}_{SW} \otimes \hat{\tau}_B \hat{U}^\dagger$, such that the evolution operator is the energy-conserving and translationally-invariant unitary, i.e. it satisfies the following commutation relations:[2,3]

$$[\hat{U}, \hat{H}_S + \hat{H}_B + \hat{H}_W] = 0, \; [\hat{U}, \hat{\Delta}_W] = 0, \tag{6}$$

where $\hat{H}_k$ is a free Hamiltonian of $k = S, B, W$ subsystem and $\hat{\Delta}_W$ is the generator of energy shifts (5).

It was proven that unitary $\hat{U}$ obeying conditions (6) can be always written in the form:[3,4]

$$\hat{U} = \hat{S}^\dagger (\hat{V}_{SB} \otimes \mathbb{1}_W) \hat{S}, \tag{7}$$

where $\hat{V}_{SB}$ is some unitary acting on the system and bath Hilbert space, $\mathbb{1}_W$ is the identity operator acting on the weight, and $\hat{S}$ is a kind of *control-shift operator* defined as follows:

$$\hat{S} = e^{-i(\hat{H}_S + \hat{H}_B) \otimes \hat{\Delta}_W} = \sum_{i,j} |\varepsilon_i\rangle\langle\varepsilon_i|_S \otimes |\varepsilon_j\rangle\langle\varepsilon_j|_B \otimes \hat{\Gamma}_{\varepsilon_i + \varepsilon_j}, \tag{8}$$

where $|\varepsilon_i\rangle_S$ is an eigenstate of the system Hamiltonian $\hat{H}_S$ and $|\varepsilon_j\rangle_B$ is an eigenstate of the $\hat{H}_B$.

Supplementary Note 3 – Control-marginal state

We define the so-called *control-marginal state*:[4]

$$\hat{\sigma}_{SB} = \mathrm{Tr}_W[\hat{S}\hat{\rho}_{SBW}\hat{S}^\dagger]. \tag{9}$$

Now, we would like to derive an alternative form of the control-marginal operator $\hat{\sigma}_{SB}$ for the product states. Firstly, for a product state $\hat{\rho}_{SBW} = \hat{\rho}_S \otimes \hat{\tau}_B \otimes \hat{\rho}_W$, we have

$$\begin{aligned}\hat{\sigma}_{SB} &= \sum_{i,j,k,l} \mathrm{Tr}_W[\hat{\Gamma}_{\varepsilon_i+\varepsilon_k}\hat{\rho}_W\hat{\Gamma}^\dagger_{\varepsilon_j+\varepsilon_l}] \,|\varepsilon_i\rangle\langle\varepsilon_i|_S\,\hat{\rho}_S\,|\varepsilon_j\rangle\langle\varepsilon_j|_S \otimes |\varepsilon_k\rangle\langle\varepsilon_k|_B\,\hat{\tau}_B\,|\varepsilon_l\rangle\langle\varepsilon_l|_B \\ &= \sum_{i,j} \mathrm{Tr}_W[\hat{\Gamma}_{\varepsilon_i}\hat{\rho}_W\hat{\Gamma}^\dagger_{\varepsilon_j}]\,|\varepsilon_i\rangle\langle\varepsilon_i|_S\,\hat{\rho}_S\,|\varepsilon_j\rangle\langle\varepsilon_j|_S \otimes \hat{\tau}_B = \mathrm{Tr}_W[e^{-i\hat{H}_S\otimes\hat{\Delta}_W}\,\hat{\rho}_S\otimes\hat{\rho}_W\,e^{i\hat{H}_S\otimes\hat{\Delta}_W}]\otimes\hat{\tau}_B \equiv \hat{\sigma}_S\otimes\hat{\tau}_B.\end{aligned} \tag{10}$$

Then, let us represent the density matrix of the weight in the time states basis, i.e. $\hat{\rho}_W = \int dt\ ds\ |t\rangle\langle t|_W\,\hat{\rho}_W\,|s\rangle\langle s|_W$. Putting it into the above formula we obtain:

$$\begin{aligned}\hat{\sigma}_S &= \int dt\ ds \sum_{i,j} \mathrm{Tr}_W[\hat{\Gamma}_{\varepsilon_i}\,|t\rangle\langle t|_W\,\hat{\rho}_W\,|s\rangle\langle s|_W\,\hat{\Gamma}^\dagger_{\varepsilon_j}]\,|\varepsilon_i\rangle\langle\varepsilon_i|_S\,\hat{\rho}_S\,|\varepsilon_j\rangle\langle\varepsilon_j|_S \\ &= \int dt\ ds \sum_{i,j} \mathrm{Tr}_W[e^{-i\varepsilon_i t}\,|t\rangle\langle t|_W\,\hat{\rho}_W\,|s\rangle\langle s|_W\,e^{i\varepsilon_j s}]\,|\varepsilon_i\rangle\langle\varepsilon_i|_S\,\hat{\rho}_S\,|\varepsilon_j\rangle\langle\varepsilon_j|_S = \int dt\ p(t)\ e^{-i\hat{H}_S t}\hat{\rho}_S e^{i\hat{H}_S t},\end{aligned} \tag{11}$$

where $p(t) = \mathrm{Tr}_W[\hat{\rho}_W\,|t\rangle\langle t|_W]$.

Supplementary Note 4 – Optimal work extraction

From the Eq. (7) further follows that work is equal to:

$$\begin{aligned}W &= \mathrm{Tr}\big[\hat{H}_W(\hat{U}\hat{\rho}_{SBW}\hat{U}^\dagger - \hat{\rho}_{SBW})\big] = \mathrm{Tr}\big[\hat{H}_{SB}(\hat{\rho}_{SBW} - \hat{U}\hat{\rho}_{SBW}\hat{U}^\dagger)\big] = \mathrm{Tr}\Big[\hat{H}_{SB}(\hat{\rho}_{SBW} - \hat{S}^\dagger\hat{V}_{SB}\hat{S}\hat{\rho}_{SBW}\hat{S}^\dagger\hat{V}^\dagger_{SB}\hat{S})\Big] \\ &= \mathrm{Tr}\Big[\hat{H}_{SB}(\hat{S}\hat{\rho}_{SBW}\hat{S}^\dagger - \hat{V}_{SB}\hat{S}\hat{\rho}_{SBW}\hat{S}^\dagger\hat{V}^\dagger_{SB})\Big] = \mathrm{Tr}\Big[\hat{H}_{SB}(\hat{\sigma}_{SB} - \hat{V}_{SB}\hat{\sigma}_{SB}\hat{V}^\dagger_{SB})\Big],\end{aligned} \tag{12}$$

where the first line follows from the energy-conservation $[\hat{U}, \hat{H}_{SB} + \hat{H}_W] = 0$ and we also used a fact that $[\hat{S}, \hat{H}_{SB}] = 0$. According to this relation, one can further straightforwardly formulate the following proposition defining the optimal work.

Proposition 1. *For arbitrary transition $\hat{\rho}_S \to \hat{\rho}'_S = \mathrm{Tr}_{BW}[\hat{U}\hat{\rho}_{SW}\otimes\hat{\tau}_B\hat{U}^\dagger]$ the work extracted by the weight is equal to:*

$$W = R(\hat{\sigma}_S\otimes\hat{\tau}_B) - R(\hat{V}_{SB}\hat{\sigma}_S\otimes\hat{\tau}_B\hat{V}^\dagger_{SB}). \tag{13}$$

Moreover, there exist a unitary $\hat{V}_{SB}$ such that $R(\hat{V}_{SB}\hat{\sigma}_S\otimes\hat{\tau}_B\hat{V}^\dagger_{SB}) = 0$, and then we have:

$$W \le R(\hat{\sigma}_S\otimes\hat{\tau}_B). \tag{14}$$

Proof. According to Eq. (12) we have

$$\begin{aligned}W &= \mathrm{Tr}\Big[\hat{H}_{SB}(\hat{\sigma}_{SB} - \hat{V}_{SB}\hat{\sigma}_{SB}\hat{V}^\dagger_{SB})\Big] - \min_{\hat{U}-\text{unitary}} \mathrm{Tr}\big[\hat{H}_{SB}\hat{U}\hat{\sigma}_{SB}\hat{U}^\dagger\big] + \min_{\hat{U}-\text{unitary}} \mathrm{Tr}\big[\hat{H}_{SB}\hat{U}\hat{\sigma}_{SB}\hat{U}^\dagger\big] \\ &= \max_{\hat{U}-\text{unitary}} \mathrm{Tr}\big[\hat{H}_{SB}(\hat{\sigma}_{SB} - \hat{U}\hat{\sigma}_{SB}\hat{U}^\dagger)\big] + \min_{\hat{U}-\text{unitary}} \mathrm{Tr}\Big[\hat{H}_{SB}(\hat{U}\hat{\sigma}_{SB}\hat{U}^\dagger - \hat{V}_{SB}\hat{\sigma}_{SB}\hat{V}^\dagger_{SB})\Big] \\ &= R(\hat{\sigma}_{SB}) - \max_{\hat{U}-\text{unitary}} \mathrm{Tr}\Big[\hat{H}_{SB}(\hat{V}_{SB}\hat{\sigma}_{SB}\hat{V}^\dagger_{SB} - \hat{U}\hat{V}_{SB}\hat{\sigma}_{SB}\hat{V}^\dagger_{SB}\hat{U}^\dagger)\Big] = R(\hat{\sigma}_{SB}) - R(\hat{V}_{SB}\hat{\sigma}_{SB}\hat{V}^\dagger_{SB})\end{aligned} \tag{15}$$

The second part follows from the fact that the arbitrary state $\hat{\sigma}_{SB}$ can be unitarily transformed to the passive state (i.e. with zero ergotropy). □

Supplementary Note 5 – Ergotropy and free energy

Proposition 2. *Let $\hat{\rho}_S$ and $\hat{\xi}_S$ are arbitrary quantum states and $\hat{\tau}_B$ is the Gibbs state. Further, $\hat{\rho}_p$ and $\hat{\xi}_p$ are passive states (i.e. with minimal energy) obtained through the unitary channel from $\hat{\rho}_S \otimes \hat{\tau}_B$ and $\hat{\xi}_S \otimes \hat{\tau}_B$, respectively. Then, if $F(\hat{\xi}_p) \le F(\hat{\rho}_p)$ it implies that*

$$R(\hat{\rho}_S \otimes \hat{\tau}_B) - R(\hat{\xi}_S \otimes \hat{\tau}_B) \le F(\hat{\rho}_S) - F(\hat{\xi}_S). \tag{16}$$

Proof. From a definition of the free energy and assumption $F(\hat{\xi}_p) \le F(\hat{\rho}_p)$ we obtain:

$$E(\hat{\xi}_p) - E(\hat{\rho}_p) - T[S(\hat{\xi}_p) - S(\hat{\rho}_p)] \le 0. \tag{17}$$

Further, we have $S(\hat{\rho}_p) = S(\hat{\rho}_S \otimes \hat{\tau}_B)$ and $S(\hat{\xi}_p) = S(\hat{\xi}_S \otimes \hat{\tau}_B)$ such that the above inequality can be rewritten in the form:

$$E(\hat{\xi}_p) - E(\hat{\xi}_S \otimes \hat{\tau}_B) - E(\hat{\rho}_p) + E(\hat{\rho}_S \otimes \hat{\tau}_B) \le F(\hat{\rho}_S \otimes \hat{\tau}_B) - F(\hat{\xi}_S \otimes \hat{\tau}_B). \tag{18}$$

Finally, since $F(\hat{\rho}_S \otimes \hat{\tau}_B) - F(\hat{\xi}_S \otimes \hat{\tau}_B) = F(\hat{\rho}_S) - F(\hat{\xi}_S)$ and $R(\hat{\rho}_S \otimes \hat{\tau}_B) = E(\hat{\rho}_S \otimes \hat{\tau}_B) - E(\hat{\rho}_p)$ (and the same for $\hat{\xi}_S$) we obtain inequality (16). □

Corollary 2.1. *For arbitrary state $\hat{\rho}_S$ and arbitrary Gibbs state $\hat{\tau}_B$ it is valid:*

$$R(\hat{\rho}_S \otimes \hat{\tau}_B) \le F(\hat{\rho}_S) - F(\hat{\tau}_S). \tag{19}$$

Proof. Let us take the state $\hat{\xi}_S$ as a Gibbs state in the same temperature as $\hat{\tau}_B$, i.e. $\hat{\xi}_S = \hat{\tau}_S$, then $\hat{\xi}_p = \hat{\tau}_S \otimes \hat{\tau}_B = \hat{\xi}_S \otimes \hat{\tau}_B$. Moreover, for arbitrary state $\hat{\rho}_S$ it is satisfied an inequality $F(\hat{\rho}_p) \ge F(\hat{\xi}_p)$, since $\hat{\xi}_p$ is a Gibbs state for which free energy has minimum. Finally, since $R(\hat{\tau}_S \otimes \hat{\tau}_B) = 0$ and from inequality (16) follows what was to be shown. □

Supplementary Note 6 – Locked energy in coherences

Proposition 3. *For the quantum state $\hat{\rho}_S$ and its control-marginal state $\hat{\sigma}_S$ (11) we have $E(\hat{\sigma}_S \otimes \hat{\tau}_B) = E(\hat{\rho}_S \otimes \hat{\tau}_B)$ and $P(\hat{\sigma}_S \otimes \hat{\tau}_B) \ge P(\hat{\rho}_S \otimes \hat{\tau}_B)$ from which follows that*

$$\Delta_C(\hat{\rho}_S, \hat{\rho}_W, \hat{\tau}_B) = R(\hat{\rho}_S \otimes \hat{\tau}_B) - R(\hat{\sigma}_S \otimes \hat{\tau}_B) \ge 0. \tag{20}$$

Proof. Firstly, let us show that the average energy of the control-marginal state is equal to the marginal state, namely

$$\begin{aligned} E(\hat{\sigma}_S \otimes \hat{\tau}_B) &= \mathrm{Tr}\left[(\hat{H}_S + \hat{H}_B)\hat{\sigma}_S \otimes \hat{\tau}_B\right] = \int dt\ p(t)\,\mathrm{Tr}\left[(\hat{H}_S + \hat{H}_B)\hat{U}_t \hat{\rho}_S \hat{U}_t^\dagger \otimes \hat{\tau}_B\right] = \\ &= \int dt\ p(t)\,\mathrm{Tr}\left[(\hat{U}_t^\dagger \hat{H}_S \hat{U}_t + \hat{H}_B)\hat{\rho}_S \otimes \hat{\tau}_B\right] = \mathrm{Tr}\left[(\hat{H}_S + \hat{H}_B)\hat{\rho}_S \otimes \hat{\tau}_B\right] = E(\hat{\rho}_S \otimes \hat{\tau}_B). \end{aligned} \tag{21}$$

Secondly, the passive energy obeys the following inequality:

$$\begin{aligned} P(\hat{\sigma}_S \otimes \hat{\tau}_B) &= \min_{\hat{V}_{SB}} \mathrm{Tr}\left[\hat{V}_{SB}^\dagger (\hat{H}_S + \hat{H}_B)\hat{V}_{SB} \hat{\sigma}_S \otimes \hat{\tau}_B\right] = \min_{\hat{V}_{SB}} \int dt\ p(t)\,\mathrm{Tr}\left[\hat{U}_t^\dagger \hat{V}_{SB}^\dagger (\hat{H}_S + \hat{H}_B)\hat{V}_{SB} \hat{U}_t \hat{\rho}_S \otimes \hat{\tau}_B\right] \\ &\ge \int dt\ p(t) \min_{\hat{V}_{SB}} \mathrm{Tr}\left[\hat{U}_t^\dagger \hat{V}_{SB}^\dagger (\hat{H}_S + \hat{H}_B)\hat{V}_{SB} \hat{U}_t \hat{\rho}_S \otimes \hat{\tau}_B\right] = \int dt\ p(t) P(\hat{\rho}_S \otimes \hat{\tau}_B) = P(\hat{\rho}_S \otimes \hat{\tau}_B). \end{aligned} \tag{22}$$

Finally, we obtain

$$R(\hat{\sigma}_S \otimes \hat{\tau}_B) = E(\hat{\sigma}_S \otimes \hat{\tau}_B) - P(\hat{\sigma}_S \otimes \hat{\tau}_B) \le E(\hat{\rho}_S \otimes \hat{\tau}_B) - P(\hat{\rho}_S \otimes \hat{\tau}_B) = R(\hat{\rho}_S \otimes \hat{\tau}_B). \tag{23}$$

□

References

1. Allahverdyan, A. E., Balian, R. & Nieuwenhuizen, T. M. Maximal work extraction from finite quantum systems. *Europhys. Lett. (EPL)* **67**, 565–571, DOI: 10.1209/epl/i2004-10101-2 (2004).
2. Skrzypczyk, P., Short, A. J. & Popescu, S. Work extraction and thermodynamics for individual quantum systems. *Nat. Commun.* **5**, 4185, DOI: 10.1038/ncomms5185 (2014).
3. Alhambra, A. M., Masanes, L., Oppenheim, J. & Perry, C. Fluctuating work: From quantum thermodynamical identities to a second law equality. *Phys. Rev. X* **6**, 041017, DOI: 10.1103/PhysRevX.6.041017 (2016).
4. Łobejko, M., Mazurek, P. & Horodecki, M. Thermodynamics of Minimal Coupling Quantum Heat Engines. *Quantum* **4**, 375, DOI: 10.22331/q-2020-12-23-375 (2020).
